# Anaemia and quality of life in chronic kidney disease: a consensus document from the European Anaemia of CKD Alliance

**DOI:** 10.1093/ckj/sfae205

**Published:** 2024-07-04

**Authors:** Indranil Dasgupta, Corinne Isnard Bagnis, Matteo Floris, Hans Furuland, Daniel Gallego Zurro, Loreto Gesualdo, Nathalie Heirman, Roberto Minutolo, Antonello Pani, José Portolés, Christian Rosenberger, José Emilio Sánchez Alvarez, Pablo Ureña Torres, Raymond C Vanholder, Christoph Wanner, European Anaemia of aCKD Alliance, Indranil Dasgupta, Corinne Isnard Bagnis, Matteo Floris, Hans Furuland, Daniel Gallego Zurro, Loreto Gesualdo, Roberto Minutolo, Antonello Pani, José Portolés, Christian Rosenberger, José Emilio Sánchez Alvarez, Pablo Ureña Torres, Raymond C Vanholder, Christoph Wanner

**Affiliations:** University Hospitals of Birmingham NHS Foundation Trust, Birmingham, UK; Warwick Medical School, University of Warwick, West Midlands, UK; Pitié Salpêtrière Hospital, APHP Sorbonne University, Paris, France; Department of Nephrology, Dialysis, and Transplantation, ARNAS G. Brotzu, Cagliari, Italy; Department of Medical Sciences, Nephrology Unit, Uppsala University Hospital, Uppsala, Sweden; European Kidney Patient's Federation, Vienna, Austria; Department of Precision and Regenerative Medicine and Ionian Area, Nephrology and Urology Units, University of Bari Aldo Moro, Bari, Italy; GSK, Waver, Belgium; Department of Advanced Medical and Surgical Sciences, University of Campania ‘Luigi Vanvitelli’, Naples, Italy; Department of Nephrology, Dialysis, and Transplantation, ARNAS G. Brotzu, Cagliari, Italy; Department of Medical Science and Public Health, University of Cagliari, Cagliari, Italy; Nephrology Department, University Hospital Puerta de Hierro, Madrid, Spain; Anaemia Working Group of S.E.N; Nephrology and Medical Intensive Care, Charité-Universitaetsmedizin Berlin, Berlin, Germany; University Hospital de Cabueñes, Asturias, Spain; Department of Nephrology and Dialysis, AURA Saint Ouen-sur-Seine, Paris, France; Department of Renal Physiology, Necker Hospital, University of Paris Descartes, Paris, France; Department of Internal Medicine and Pediatrics, Nephrology Section, University Hospital, Ghent, Belgium; European Kidney Health Alliance, Brussels, Belgium; Department of Clinical Research and Epidemiology, Comprehensive Heart Failure Centre, University of Würzburg, Würzburg, Germany

**Keywords:** anaemia, CKD, dialysis, guidelines, quality of life

## Abstract

Anaemia is common in chronic kidney disease (CKD) and has a significant impact on quality of life (QoL), work productivity and outcomes. Current management includes oral or intravenous iron and erythropoiesis-stimulating agents (ESAs), to which hypoxia-inducible factor prolyl hydroxylase inhibitors (HIF-PHIs) have been recently added, increasing the available therapeutic options. In randomised controlled trials, only intravenous iron improved cardiovascular outcome, while some ESAs were associated with increased adverse cardiovascular events. Despite therapeutic advances, several challenges and unmet needs remain in the current management of anaemia of CKD. In particular, clinical practice does not include an assessment of QoL, which prompted a group of European nephrologists and representatives of patient advocacy groups to revisit the current approach. In this consensus document, the authors propose a move towards a more holistic, personalised and long-term approach, based on existing evidence. The focus of treatment should be on improving QoL without increasing the risk of adverse cardiovascular events, and tailoring management strategies to the needs of the individual. In addition, the authors discuss the suitability of a currently available anaemia of CKD–specific health-related QoL measure for inclusion in the routine clinical management of anaemia of CKD. The authors also outline the logistics and challenges of incorporating such a measure into electronic health records and how it may be used to improve QoL for people with anaemia of CKD.

## IMPACT OF ANAEMIA IN CHRONIC KIDNEY DISEASE (CKD)

Anaemia is a common complication of CKD that has a significant humanistic and societal impact, in particular, a negative impact on the quality of life (QoL) of people with CKD and their caregivers [1–3]. Therefore, it is imperative that people with CKD are regularly assessed and treated for anaemia [4]. The prevalence and severity of anaemia increases as kidney function declines, with up to 60% of people with non-dialysis-dependent CKD having anaemia [5]. Anaemia is more common and occurs earlier in people with CKD who have diabetes [6], one of the leading causes of CKD [7]. The causes of anaemia of CKD are multifactorial and include reduced production of endogenous erythropoietin, absolute and/or functional iron deficiency, inflammation and subclinical blood loss, among others [8].

In people with CKD, worsening of anaemia results in a poor clinical outcome with wide-reaching effects. Anaemia can lead to a reduction in work productivity [2] and impact patient physical functioning (e.g. fatigue), emotional state (e.g. feeling sad or depressed), daily activities (e.g. taking care of their family) [9], self-esteem [10] and sexual function [10, 11]. The main symptoms of anaemia of CKD that impair QoL are fatigue and shortness of breath [12]. Anaemia of CKD is also associated with worsening angina, impaired cardiac contractile function and left ventricular hypertrophy, which can result in increased hospitalizations and mortality [13, 14], decreased functional capacity and increased risk of falls in elderly people [15].

The European Anaemia of CKD Alliance was convened by a group of concerned nephrologists and patient association representatives in December 2022 (see Acknowledgements) to highlight the needs of people with anaemia of CKD and optimize disease management to improve their QoL and outcomes. The participants proposed a 7-point consensus document (Table 1) of actions needed for a more efficient and practical approach for the community involved in kidney care. The current article is an elaboration of that consensus document.

**Table 1:** Consensus document on the actions needed for a more efficient and practical approach for the community involved in kidney care.

We, the European Anaemia of CKD Alliance, are committed to raising awareness and challenging ourselves and others to think differently about the long-term management of anaemia of CKD for the benefit of people affected.	
1	Anaemia severely affects QoL in people with CKD [1, 2, 12], often impairing people's usual daily activities; QoL should be considered carefully at each clinic visit.
2	Anaemia of CKD is associated with the risk of major cardiovascular events, hospitalisation and death [24, 25], which should be balanced against an overall increase in cardiovascular risk associated with ESA therapy.
3	Physicians need tools and techniques to fully appreciate the impact of anaemia on everyday life; evidence suggests that anaemia of CKD is inadequately treated across Europe [26].
4	Iron therapy is important but underutilised in practice, which may contribute to the suboptimal management of anaemia of CKD and its continued negative impact on QoL.
5	Anaemia is partially corrected in most people with CKD; there is a need to treat it more effectively and with a greater sense of urgency to reduce the impact of its symptoms on peoples’ lives.
6	Developing and communicating the evidence in support of personalised management, patient engagement and expansion of treatment options may help advance the treatment of people with symptomatic anaemia of CKD.
7	We advocate a shift towards a holistic, personalised, evidence-based, long-term management approach in which patients are fully informed of their treatment options and the positive and negative effects of treatments, which will allow people with anaemia of CKD to make informed, shared decisions with their physician to best suit their needs.

The alliance comprised 19 members from across Europe, including 17 nephrologists with an interest in anaemia of CKD, and representatives of the European Kidney Patient Federation, Kidney Care UK (including a patient advocate) and the European Kidney Health Alliance. Several meetings were conducted by an independent, external facilitator and the methodology included individual touch points with the members, small working group sessions and board meetings. During these meetings the external facilitator gathered advice and expertise from the members to create the manifesto, bringing together the views of the different stakeholders (patients, policymakers and nephrologists) involved in the management of anaemia of CKD.

## CURRENT MANAGEMENT OF ANAEMIA OF CKD

The key treatments for managing anaemia of CKD are oral or intravenous (IV) iron and erythropoiesis-stimulating agents (ESAs) [8]. Recently, hypoxia-inducible factor prolyl hydroxylase inhibitors (HIF-PHIs) have been added to the therapeutic armamentarium and have demonstrated a similar efficacy and tolerability profile to ESAs [16–19]. As oral agents, HIF-PHIs are a potential treatment option for those who are intolerant to ESAs, have needle phobia or are receiving home-based dialysis. Further, HIF-PHIs have a beneficial effect on iron metabolism similar to ESAs and, as such, may potentially reduce the need for IV iron infusion [17]. Several HIF-PHIs have received regulatory approval and are likely to be used increasingly in the future [16–19]. However, regulatory approval differs between the USA and Europe depending on the target patient groups.

Treatment of anaemia of CKD is guided by data from randomised trials of ESAs, which demonstrated that normalisation of haemoglobin (Hb) levels (13.0–15.0 g/dl) did not reduce cardiovascular events compared with a lower target range (10.5–11.5 g/dl) [20]. In contrast, overcorrection of Hb beyond a certain range in people treated with ESAs was associated with an increased risk of cardiovascular events, thrombotic episodes, hospitalisation and mortality in some of these trials [21–23]. Most of these trials did not directly examine the impact of Hb correction on QoL. A critical look at the large ESA trials, particularly in people with non-dialysis-dependent CKD, suggests that the risk:benefit ratio between adverse events and QoL gains may be acceptable (Table 2).

**Table 2:** Hb correction and QoL outcomes from anaemia trials.

Trial name	Study design	QoL outcomes	References
ASCEND-NHQ (NCT03409107)	A multicentre, randomised, double-blind, placebo-controlled trial was carried out in 142 centres across 14 countries and consisted of 4 weeks of screening, 28 weeks of treatment, with a follow-up at 4–6 weeks	ASCEND-NHQ demonstrated that daprodustat (*n* = 307) was superior to placebo (*n* = 307) in increasing Hb levels among adults with CKD stages 3–5 not receiving dialysis. Greater improvements in fatigue were also shown for patients receiving daprodustat compared with placebo. The mean change in SF-36 score was also higher at week 28 in patients receiving daprodustat than those who received placebo.	[34]
CHOIR (NCT00211120)	A randomised, open-label trial conducted across 130 centres in the USA. The median study duration was 16 months	The CHOIR trial showed an increased risk of cardiovascular events and no improvement in QoL for adult patients receiving dialysis treated to a Hb target of 13.5 g/dl (*n*= 715) compared with those treated to a lower target of 11.3 g/dl (*n* = 717).	[22]
CREATE (NCT00321919)	A randomised, open-label, parallel-group trial was conducted across 94 centres in 22 countries. The mean time of observation for the primary endpoint was 3 years	The CREATE trial demonstrated improved QoL without an increased risk of cardiovascular events in adults with CKD randomised to a higher Hb target (13.0–15.0 g/dl) (*n* = 301), despite >90% of patients having cardiovascular morbidities at baseline.	[20]
Iron and Heart (EudraCT: 2014-004133-16)	A prospective, multicentre, randomised, double-blind trial was carried out in seven centres in the UK over 12 weeks	The Iron and Heart trial showed that in non-anaemic adults with stage 3b–5 CKD and iron deficiency, not receiving dialysis, IV iron maintained a stable Hb concentration at months 1 and 3 (*n* = 26) compared with placebo (*n* = 28). A modest, numerical improvement in QoL and functional capacity was observed.	[35–37]
Iron and Muscle (EudraCT: 2018-000144-25)	A prospective, multicentre, randomised, double-blind trial in the UK over 12 weeks	The Iron and Muscle trial showed that in patients with non-anaemic stage 3b–5 CKD and iron deficiency not receiving dialysis, there was no significant impact of IV iron (*n* = 38) versus placebo (*n* = 37) on exercise capacity, functional capacity or QoL.	[35, 37]
FIND-CKD (NCT00994318)	A prospective, multicentre, randomised, open-label, 56-week trial conducted in 193 centres across 20 countries	The FIND-CKD trial randomised adult patients with non-dialysis-dependent CKD, anaemia and iron deficiency to receive high-ferritin IV iron (*n* = 155), low-ferritin IV iron (*n* = 154) or oral iron (*n* = 317). Patients treated with higher ferritin quickly reached and maintained the Hb target (increase ≥1 g/dl) and were less likely to require ESA treatment compared with the other treatment arms. No significant differences in QoL outcomes were observed between the treatment arms.	[38]
PIVOTAL (EudraCT: 2013-002267-25)	A randomised, open-label, blind-endpoint controlled trial and post hoc analysis carried out in 50 centres across the UK. The median follow-up was 2.1 years	In the PIVOTAL trial of IV iron therapy in adult patients undergoing haemodialysis, there were lower cardiovascular event and mortality rates in the proactive (high-dose IV iron; *n* = 1093) arm compared with the reactive arm (low-dose IV iron; *n* = 1048). Further analysis of the baseline data of the PIVOTAL trial (*n* = 2141) showed that QoL at baseline was low; transferrin saturation ≤20% was associated with a worse physical component score of QoL and lower QoL at baseline was predictive of all-cause mortality and cardiovascular events.	[32, 39]
TREAT (NCT00093015)	A post hoc analysis of a randomised, double-blind, placebo-controlled trial conducted in 623 centres across 24 countries over 97 weeks	A post hoc analysis of the TREAT trial (*n* = 4038) in adults with diabetes and non-dialysis CKD and anaemia demonstrated small but consistent improvement in fatigue and overall QoL in the darbepoetin alfa–treated group compared with placebo.	[40]

CKD: chronic kidney disease; Hb, haemoglobin; IV, intravenous; SF-36, Study 36-item Short-Form Health Survey; QoL: quality of life; UK, United Kingdom; US, United States.

The clinical management guidelines for anaemia of CKD have generally been steered by measurable clinical outcomes rather than the needs or QoL of individuals. The Kidney Disease: Improving Global Outcomes (KDIGO) guidelines advise against starting ESAs when Hb levels are ≥10.0 g/dl, using ESA to maintain Hb >11.5 g/dl and intentionally increasing Hb to >13 g/dl [8], but suggest aiming for a higher Hb level in individual patients to improve QoL if the benefits outweigh the risks [8]. The European Renal Best Practice (ERBP) position statement suggests that in low-risk patients (e.g. young patients with very few comorbidities) or those likely to benefit in terms of QoL, ESA therapy may be started at a higher Hb value [27]. The guidelines also recommended that for people at risk of cardiovascular events, such as those with diabetes or heart disease or those hyporesponsive to ESA treatment, the aim should be to target a lower Hb range (10–12 g/dl) [27].

## CHALLENGES AND UNMET NEEDS IN THE MANAGEMENT OF ANAEMIA OF CKD

**Figure 1:**
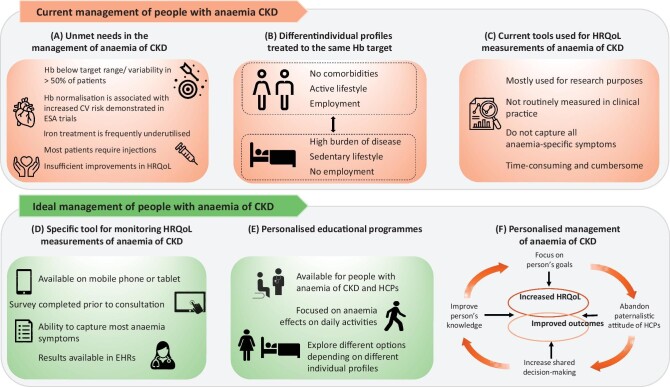
Summary of QoL issues for people with anaemia of CKD. The current management of people with anaemia of CKD and its impact on QoL is illustrated in the upper panels in terms of **(A)** unmet needs in the management of anaemia of CKD, **(B)** opposing individual profiles and **(C)** current tools used for HRQoL measurements in anaemia of CKD. The ideal management of people with anaemia of CKD is illustrated in the lower panel with a focus on **(D)** the ideal tool for monitoring HRQoL measurements in anaemia of CKD, **(E)** personalised anaemia of CKD educational programs and **(F)** personalised management of anaemia of CKD. CV: cardiovascular.

Despite advancements in the management of anaemia of CKD over the past 3 decades, there remain significant challenges and unmet needs (Fig. 1A–C):

1. Most people with advanced CKD not on dialysis fail to maintain Hb targets in the medium-to-long term [26]. Hb instability in CKD is associated with an increased risk of mortality [28–30].
2. A single target range of Hb may not apply to all people with CKD, as there is significant variability in Hb levels due to age, sex, geography, aetiology of kidney disease and estimated glomerular filtration rate [31].
3. Hb normalisation and rapid correction of anaemia are avoided because of the increased risk of cardiovascular events and vascular access thrombosis, as demonstrated in large ESA trials, although results were not granular enough to identify the factors responsible for this (Table 2).
4. Despite the demonstrated benefits of increasing Hb levels to targets and clinical outcomes [32], parenteral iron is underutilised due to the perceived adverse effects and administration difficulties [33].
5. Administration of ESA and IV iron in people who are not treated by haemodialysis often requires assistance from a healthcare professional (HCP) or hospital attendance by the patient, increasing healthcare burden and cost.
6. There is no consistent policy pursuing a meaningful improvement in patient-reported outcomes and the health-related quality of life (HRQoL) of people with anaemia of CKD.

## HOW DO WE ADDRESS THE CHALLENGES AND UNMET NEEDS?

First, education programs are needed to provide people with anaemia of CKD and their caregivers with information on the condition, its impact on HRQoL and daily activities and management strategies. Educational tools should be co-created with patients and be in lay language, with features that allow the patient to add notes, questions and concerns prior to their consultation. In addition, specific measures are needed to reach more difficult-to-contact people, such as migrants, minorities, people who are unable to use technology, adolescents and older people. Once appropriately implemented, artificial intelligence (AI) could be used to accurately translate educational tools into different languages to accommodate people from diverse regions in the future. Furthermore, AI could validate language translations to ensure that the meaning is retained. This will help engage patients and empower them to discuss the most appropriate management strategies and treatment for their symptoms with their HCP when they attend clinical consultations.

The intensity of treatment for anaemia of CKD and target Hb levels should be based on age, gender, primary renal disease, comorbidities, employment and activity status and personal expectations of QoL. For example, the needs of someone without significant comorbidity who has a young family, is employed full time and has a very active lifestyle are completely different from those of an age-matched individual with multiple cardiovascular comorbidities and a sedentary lifestyle. However, most patients lie somewhere in between these two extremes, requiring careful consideration of the different elements contributing to decision-making and dialogue with the patient. Further, the individual preference to use either injectable or oral preparations should also be considered. National Institute for Health and Care Excellence clinical guidelines recommend that patients should be informed of their choices and be involved in decisions about their care [41]. These observations call for personalised management that encourages shared decision-making [41] rather than a blanket approach to target Hb range and/or a specific ESA for everyone.

For people with symptomatic anaemia of CKD, particularly fatigue, improved HRQoL is arguably the most important objective of anaemia management [41–43]. A cross-sectional analysis of a large European CKD patient survey found significant correlation between Hb level and HRQoL impairment, irrespective of the instrument used [1]. The people with CKD and anaemia typically had a consistently lower HRQoL than those without anaemia, suggesting a significant contribution of anaemia itself. Impaired HRQoL was more apparent in people not on dialysis with stage 3 or 4 CKD than those who were on dialysis [1]. However, the effect of anaemia treatment on QoL is not routinely assessed in clinical practice. We believe it is crucial to measure HRQoL as the first step towards improving the management of anaemia of CKD.

## HRQOL TOOLS FOR MANAGEMENT OF ANAEMIA OF CKD

The most commonly used HRQoL instruments in kidney disease are the 36-Item Short Form Survey (SF-36), 12-Item Short Form Survey, European Quality of Life 5 Dimensions, Patient-Reported Outcomes Measurement Information System and Kidney Disease Questionnaire [44–46]. These instruments are mainly used for research purposes, are time-consuming and cumbersome and do not capture all symptoms of anaemia of CKD or the potential impact of anaemia treatment on HRQoL. For example, the SF-36 does not measure sleep disturbances or cognitive impairment [45]. Therefore, there is a need for a questionnaire that is specific to HRQoL of anaemia of CKD, will capture most of the symptoms of anaemia of CKD and is suitable for use in nephrology clinics without impacting consultation time. Ideally, these existing instruments should be supported by digital tools.

In 2020, a new, anaemia-specific HRQoL questionnaire containing 23 items, the Chronic Kidney Disease and Anaemia Questionnaire (CKD-AQ), was developed and later updated to version 2, containing 21 items in 2022 [12, 47]. The design was based on qualitative concept elicitation and cognitive debriefing interviews with people with anaemia of CKD to assess the frequency, duration, severity and impact of their symptoms [12, 47]. The CKD-AQ is structured into two groups of questions: the symptoms (energy, weakness, tiredness, shortness of breath during rest or activity, bruised skin and difficulty remembering) and the impact of anaemia on daily life (sleeping problems, lack of motivation, need for frequent breaks, difficulty standing for long periods, feeling distressed and feeling burdensome) [12]. The content validity of the CKD-AQ was assessed in three rounds of interviews and linguistic translation and cultural adaptation into 68 languages was carried out with the aim of using this tool in future studies and clinical practice [12]. The CKD-AQ was used alongside the SF-36 vitality score in the ASCEND-NHQ trial (NCT03409107) to evaluate improvement in QoL with daprodustat compared with placebo in people with non-dialysis-dependent CKD. Improvements in CKD-AQ symptom scores in the active arm compared with the control arm of the trial corresponded with changes in SF-36 vitality scores [34]. The CKD-AQ is quick to complete and accessible online for free, hence it has the potential to help clinicians assess the symptom burden of anaemia of CKD for the individual and evaluate treatment options as part of routine clinical care. Education programs for people with anaemia of CKD and HCPs, as mentioned previously, are needed to drive uptake of the questionnaire.

## IMPLEMENTATION OF AN ANAEMIA OF CKD–SPECIFIC HRQOL TOOL IN ROUTINE CARE

**Figure 2:**
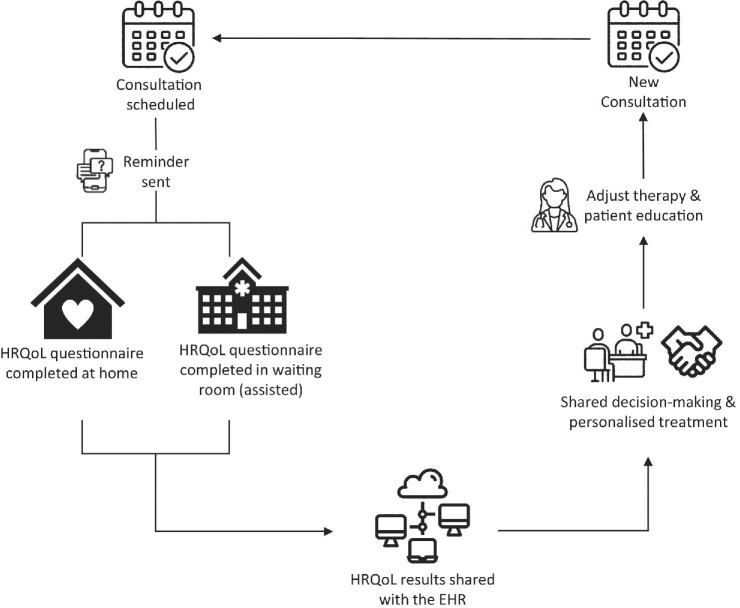
HRQoL survey management. Proposed pathway for comprehensive HRQoL management in the hospital and out of hospital.

We propose a strategy that can be adapted to cater to the individual needs of different people with anaemia of CKD and/or caregivers while considering the stage of CKD, treatment modality, time spent on completing the HRQoL questionnaire and the automatic incorporation of results into electronic health records (EHRs). We envisage that people with anaemia of CKD will complete the electronic HRQoL questionnaire themselves or be assisted by a caregiver, either at home or in the waiting room, using their own mobile phone or tablet, prior to a consultation with an HCP. The answers could be sent directly to the patient's EHR and presented to the clinician as a comprehensive summary, illustrative diagram and/or a score. The clinician would review and compare the HRQoL results with previous results, where available, and corroborate these with Hb values and other variables that may influence HRQoL. These considerations could inform shared treatment decisions with patients (Fig. 2).

There are challenges to implementing electronic patient-reported outcome measures (PROMs) such as HRQoL in routine care, including patient-, HCP- and service-level barriers [48]. For people with anaemia of CKD, the main barriers are the time required to complete the questionnaire and the inability to use electronic devices. Paper questionnaires for people who cannot use electronic devices and shortened questionnaires sent via youth social media networks (e.g. TikTok) should be considered. At the HCP level, the main barriers are insufficient time to interpret the PROMs, lack of knowledge regarding interpretation, the perceived uselessness of PROMs and difficulty in using the electronic PROM system [48]. At the service level, the main barriers are difficulty in integrating PROMs into the electronic patient management system, inability to respond to the data generated, inadequate information technology infrastructure to collect and use PROMs, the need for potential security strategies to ensure data protection and the lack of resources for implementation [48]. These barriers must be recognised and examined to understand the support and adaptations needed to overcome them (Table 3).

**Table 3:** Summary of the proposed strategies to overcome the challenges of implementing electronic HRQoL PROMs in daily clinical practice.

Challenges		
People with anaemia of CKD	HCP	Service level
Time required to complete the questionnaire	Time required to interpret and action PROMs	Difficulty integrating PROMs into the EHR
Inability to use electronic devices	Lack of knowledge to interpret and action PROMs	Inability to respond to the data generated
Perceived irrelevance of the exercise	Perceived uselessness of PROMs	Lack of resources to support effective integration
Primary concerns	Difficulty integrating PROMs into the EHR or routine practice	Lack of infrastructure to collect and interpret PROMs
How to overcome		
Acknowledgement and engagement	HCPs, MDTs and people with anaemia of CKD should understand the value of implementing PROMs in clinical practice.	
Optimal tool selection	A disease-specific PROM, such as the CKD-AQ, can be appropriate to assess the impact of anaemia on QoL and guide the individual with anaemia of CKD and the clinician on treatment choices and patient goals.	
Accessible and inclusive formats	While digital platforms may be preferred, paper-based options should be available for those who are less digitally competent. For patients with cultural, technical or physical barriers, neutral aid, such as assistance from nurses, should be available.	
Robust IT infrastructure	A reliable and secure online system for at-home PROMs could facilitate immediate access to results for the healthcare team.	
Early stakeholder engagement	There are many stakeholders involved (HCPs, patients, MDTs), each of whom should be engaged early to overcome technical hurdles and facilitate a smooth integration into routine practice.	
Data processing and discussion	How PROM data are processed, discussed and shared with other HCPs, especially the affected individual's GP, is crucial.	
Training for HCPs	Enhancing the skills of HCPs on the value and interpretation of PROMs ensures higher response rates and better engagement with people with anaemia of CKD.	
Clear communication with patients	Addressing the concerns of people with anaemia of CKD and explaining the importance of PROMs can enhance participation.	

GP: general practitioner; IT: information technology; MDT: multidisciplinary team.

To address these challenges, there are technical and infrastructure choices to consider when integrating an HRQoL questionnaire into clinical practice [49]. The questionnaire should optimize the experience of people with CKD, minimize disruption to the daily running of the clinic and enhance the clinical use of data. A number of steps are required for implementation, including willingness of both the HCP and the patient to participate in the collection of data using the questionnaire, selection of a patient-centric tool (e.g. CKD-AQ or SF-36), integration of the tool into the EHRs and technical considerations, such as how the data will be shared between the HCP and the patient (e.g. electronically or through a paper-based system) [49].

The national kidney registries provide examples of how to collect PROMs [50]. A paper-based questionnaire should be available for people who are less digitally competent. It must be determined where people will complete the questionnaire (e.g. in the clinic prior to their appointment or at home); a person on in-centre haemodialysis may prefer to complete the questionnaire during a dialysis session. The clinic should consider how to share reminders to complete the questionnaire (e.g. via email, letter or text message). How data will appear in the EHRs should also be determined (e.g. as scores or as text).

Use of the same PROM platform and questionnaires across centres would improve the interpretation of results by comparing the data with an aggregate benchmark. Furthermore, HRQoL data could be correlated with outcomes if data from PROMs are stored in renal registry databases, although this would require informed consent.

## FUTURE OUTLOOK AND CONCLUSIONS

A move towards an integrated patient management approach is needed to improve patient-centred care in anaemia of CKD, with a focus on QoL. Patients should be encouraged to engage with interactive educational materials, which may help them to understand the utility of an HRQoL instrument in managing their condition. This also offers a platform where patients with anaemia of CKD can educate themselves and actively manage their QoL (Fig. 1D–F). Integrating PROMs into the EHRs may facilitate the continuity of care, ensuring that HCPs will be regularly updated about their patient's self-reported experiences and outcomes. Since improved QoL may come at the expense of major adverse cardiovascular and kidney events, individual risk assessment is crucial.

The members of the European Anaemia of CKD Alliance advocate a shift towards a holistic, personalised, evidence-based and long-term management approach in which people with anaemia of CKD are fully informed of their treatment options and make shared decisions with their physician that best suit their individual needs and preferences. Additionally, patients should be consulted early in the process of designing large clinical trials so that outcomes important to them are considered in future trials.

## Data Availability

No new data were generated or analysed in support of this research.
